# PAY RATES AND EMOTIONAL LABOR: CONTENT ANALYSIS OF JOB ADVERTISEMENTS FOR HOME HEALTH AIDES

**DOI:** 10.1093/geroni/igac059.3026

**Published:** 2022-12-20

**Authors:** Brittney Pond

**Affiliations:** University of California, San Francisco, San Francisco, California, United States

## Abstract

In the United States, over 3.5 million home health aides provide care and assistance to older adults and people with disabilities living in the community (Almeida, Cohen, Stone, & Weller, 2021). These providers are often tasked with emotionally and physically demanding work and are poorly compensated for their labor. In this poster I discuss the connection between the emotional demands of home health care jobs and their compensation through a quantitative content analysis of online job advertisements for home health aide positions (n=312). This research addresses two primary research questions: 1) what are the prevailing pay rates for home health aides and how do they compare to living wages? 2) what is the relationship between emotion work requirements and pay rates in home health aide job advertisements? In about 57% of the job advertisements hourly wages were not sufficient to meet the living wage level according to the MIT Living Wage Calculator. Using linear probability models, I found that there is no statistically significant difference between pay rates listed by home health agencies requiring emotional labor and home health agencies not requiring emotional labor while holding education, experience, and licensure requirements constant. Stratifying the data by home health agency/company size (1,000 employees or less and 5,000 employees or more) yielded no statistically significant results. These findings support and expand upon existing literature that home health aides are undercompensated for their work regardless of agency size, and provide evidence for the need for fair compensation and support.

